# *TCF7L2* rs290481 T>C polymorphism is associated with an increased risk of type 2 diabetes mellitus and fasting plasma glucose level

**DOI:** 10.18632/oncotarget.20300

**Published:** 2017-08-16

**Authors:** Li Zhu, Zhiqiang Xie, Jianping Lu, Qiu Hao, Mingqiang Kang, Shuchen Chen, Weifeng Tang, Hao Ding, Yu Chen, Chao Liu, Haojie Wu

**Affiliations:** ^1^ Department of Nephrology, Affiliated People's Hospital of Jiangsu University, Zhenjiang, Jiangsu Province, China; ^2^ Department of Clinical Laboratory, Fujian Medical University Union Hospital, Fuzhou, Fujian Province, China; ^3^ Department of Pathology, Fujian Cancer Hospital, Fujian Medical University Cancer Hospital, Fuzhou, Fujian Province, China; ^4^ Department of Immunology, School of Medicine, Jiangsu University, Zhenjiang, Jiangsu Province, China; ^5^ Department of Thoracic Surgery, Fujian Medical University Union Hospital, Fuzhou, Fujian Province, China; ^6^ Department of Respiratory Disease, Affiliated People's Hospital of Jiangsu University, Zhenjiang, Jiangsu Province, China; ^7^ Department of Medical Oncology, Fujian Provincial Cancer Hospital, Fujian Medical University Cancer Hospital, Fuzhou, Fujian Province, China; ^8^ Department of Cardiothoracic Surgery, Affiliated People's Hospital of Jiangsu University, Zhenjiang, Jiangsu Province, China; ^9^ Department of Endocrinology, Zhongshan Hospital Xiamen University, Xiamen, Fujian Province, China

**Keywords:** TCF7L2, polymorphism, type 2 diabetes mellitus, risk

## Abstract

Genetic polymorphisms of the transcription factor 7-like 2 (*TCF7L2*) gene may be key agents in the etiology of type 2 diabetes mellitus (T2DM). In the present case-control study, we aimed to assess the possible relationship of *TCF7L2* polymorphisms with T2DM and determine the effect of *TCF7L2* polymorphisms on the level of fasting plasma glucose (FPG) in Eastern Chinese Han subjects. The *TCF7L2* rs7903146C>T and rs290481 T>C polymorphisms were genotyped by SNPscan genotyping assays in 502 subjects with T2DM and 782 non-diabetic controls. After adjusting for age, gender, drinking, smoking and body mass index (BMI), the association of *TCF7L2* rs7903146C>T and rs290481 T>C polymorphisms with T2DM was determined. We found that *TCF7L2* rs290481 T>C polymorphism increased the susceptibility of T2DM in the overall comparison. In subgroup analyses by age, sex, BMI, alcohol use and smoking status, a significantly increased risk of T2DM was also found in female, older subject and never drinking and BMI < 24 kg/m^2^ subgroups. The relationship of *TCF7L2* rs290481 T>C polymorphism with the biochemistry characteristics in controls was also assessed. We found that *TCF7L2* rs290481 T>C polymorphism significantly increased the level of FPG in controls. Our findings suggest that *TCF7L2* rs290481 T>C polymorphism is associated with T2DM in Eastern Chinese Han population and links to variations in FPG level. In addition, these relationships are more pronounced in female, older subject and never drinking and BMI < 24 kg/m^2^ subgroups. A comprehensive fine-mapping study with functional investigation is needed to confirm or refute these potential correlations.

## INTRODUCTION

Type 2 diabetes mellitus (T2DM), a chronic metabolic disease, is a common form of diabetes. Over the past decades, the incidence of T2DM in Chinese adult has increased dramatically and is about 11.6% [1]. T2DM is considered a serious public health threat. T2DM is primarily characterized by disturbance of energy metabolism, relative insulin deficiency and/or insulin resistance. Meanwhile, T2DM is a complex metabolic disorder resulting from multiple genetic components and environmental risk factors. The lipid metabolism and insulin signal transduction candidate genes may participate in the pathogenesis of T2DM. The variants of these candidate genes could change the expression of protein and also lead to abnormal signal transduction or metabolic disorder. Finally, these variants may influence the susceptibility of T2DM.

Transcription factor 7-like 2 (TCF7L2), a tran-scription factor, is a member of the high mobility group-box (HMGB) family [2] and exerting a variety of functions. TCF7L2 expresses in pancreatic β-cells [3] and plays an important role in glucose homeostasis. In addition, it is reported that TCF7L2 leads to the activation of Wnt target genes, and then it promotes the proliferation of pancreatic β-cells and the production of the incretin hormone glucagon-like peptide-1 in enteroendocrine cells [4]. Grant *et al.* reported that *TCF7L2* polymorphisms may be associated with the risk of T2DM in an Icelandic population [5]. From then on, variants of *TCF7L2* are considered as important genetic components in assessing the risk for T2DM worldwide [3, 6–8].

The *TCF7L2* gene lies in chromosome 10q25.2-q25.3. *TCF7L2* gene is polymorphic, and a number of loci have been identified, such as the rs10749127, rs10787475, rs11196224, rs12775879, rs17130188, rs290481, rs290487, rs290489, rs3750804, rs4918792, rs6585194, rs7085532, rs7094463, rs7919409, and rs966227, rs7903146, rs11196205 and rs12255372 polymorphisms, etc. Among these single nucleotide polymorphisms (SNPs), the *TCF7L2* rs7903146 polymorphism are widely studied for the potential relationship with T2DM susceptibility. Recently, several case-control studies found that *TCF7L2* rs7903146 C>T is associated with the development of T2DM [9–12]. Then, a meta-analysis suggested that *TCF7L2* rs7903146 C>T variants might confer an increased risk to T2DM in Chinese Han population [13]. However, in that meta-analysis, the number of included studies was moderate. Then, the power of the study may be limited. Several recent reports focused on the relationship of *TCF7L2* rs290481 T>C with diabetes mellitus and the results were conflicting. Genetic variations of *TCF7L2* genes, such as rs7903146C>T and rs290481 T>C, may contribute to the etiology of T2DM. Therefore, we designed a hospital-based case-control study with 502 T2DM cases and 782 healthy controls and selected *TCF7L2* rs7903146C>T and rs290481 T>C polymorphisms to explore the potential relationship of these SNPs with T2DM risk in an Eastern Chinese Han population.

## RESULTS

### Baseline characteristics

Basic clinical characteristics (e.g. anthropometric data, demographics and risk factors) and biochemical laboratory are summarized in Table 1. Locus information of *TCF7L2* rs7903146C>T and rs290481 T>C polymorphisms is listed in Table 2. All genotyping success rate for these SNPs was more than 99%. Minor allele frequency (MAF) in Chinese database and our controls is shown in Table 2. In addition, the deviations from the Hardy-Weinberg equilibrium (HWE) in controls are listed in Table 2.

**Table 1:** Distribution of selected demographic variables and risk factors in T2DM cases and controls

Variable	Cases (n=502)		Controls (n=782)		*P*^a^
	n	%	n	%	
Age (years)	65.20 (±9.51)		64.67 (±9.80)		0.347
Age (years)					0.113
< 65	227	45.22	389	49.74	
≥ 65	275	54.78	393	50.26	
Sex					0.819
Male	332	66.14	522	66.75	
Female	170	33.86	260	33.25	
Smoking status					0.264
Never	333	66.33	542	69.31	
Ever	169	33.67	240	30.69	
Alcohol use					0.263
Never	453	90.24	690	88.24	
Ever	49	9.76	92	11.76	
BMI (kg/m^2^)	24.95 (±3.64)		23.51 (±2.94)		**<0.001**
BMI (kg/m^2^)					**<0.001**
< 24	210		436		
≥ 24	292		346		
Systolic pressure (mmHg)	135.08 (±17.83)		134.02 (±17.71)		0.297
Diastolic pressure (mmHg)	79.79 (±10.35)		80.06 (±10.02)		0.649
FPG (mmol/L)	8.08 (±2.76)		5.13 (±0.49)		**<0.001**
Total cholesterol (mmol/L)	4.61 (±1.24)		4.88 (±1.02)		**<0.001**
Triglyceride (mmol/L)	1.74 (±1.14)		1.55 (±0.96)		0.001
HDL-C (mmol/L)	1.13 (±0.37)		1.30 (±0.37)		**<0.001**
LDL-C (mmol/L)	3.00 (±1.07)		3.14 (±0.82)		0.010

^a^ Two-sided *χ*^2^ test and student t test; Bold values are statistically significant (*P* <0.05); BMI, body mass index; FPG, fasting plasma glucose; HDL-C, high-density lipoprotein cholesterol; LDL-C, low-density lipoprotein cholesterol.

**Table 2:** Primary information for *TCF7L2* rs7903146C>T and rs290481 T>C polymorphisms

Genotyped SNPs	Chromosome	Chr Pos (NCBI Build 37)	Region	MAF^a^ for Chinese in database	MAF in our controls (n = 782)	*P* value for HWE^b^ test in our controls	Genotyping method	Genotyping value (%)
*TCF7L2* rs7903146 C>T	10	114758349	Intron 4	0.026	0.028	0.584	SNPscan	99.61
*TCF7L2* rs290481 T>C	10	114923825	Intron 13	0.405	0.379	0.057	SNPscan	99.30

^a^ MAF: minor allele frequency;

^b^ HWE: Hardy–Weinberg equilibrium;

### Association of *TCF7L2* rs7903146C>T and rs290481 T>C polymorphisms with T2DM

The genotype distributions of *TCF7L2* rs7903146C>T and rs290481 T>C polymorphisms are summarized in Table 3. Genotype distributions for these two SNPs were tested for HWE and no significant deviation was identified in controls (Table 2). In the analysis of *TCF7L2* rs290481 T>C SNP, differences in the distribution of the rs290481 TC genotype compared with the rs290481 TT genotype and rs290481 TC/CC genotype compared with the rs290481 TT genotype between 502 T2DM cases and 782 controls were found [TC vs. TT: crude odds ratio (OR) = 1.29, 95% confidence interval (CI) = 1.01–1.65, *P* = 0.042 and TC/CC vs. TT: crude OR = 1.27, 95% CI = 1.01–1.61, *P* = 0.044 (Table 3)]. Results of multivariate linear regression analysis demonstrated that *TCF7L2* rs290481 T>C polymorphism was correlated with the development of T2DM in two genetic models. When the *TCF7L2* rs290481 TT genotype was used as the reference group, the rs290481 TC and TC/CC genotype were correlated with the increased risk of T2DM [TC vs. TT: adjusted OR = 1.32, 95% CI = 1.03–1.69, *P* = 0.030 and TC/CC vs. TT: adjusted OR = 1.30, 95% CI = 1.02–1.64, *P* = 0.032 (Table 3)]. However, we found that *TCF7L2* rs7903146C>T polymorphism was not associated with the development of T2DM (Table 3).

**Table 3:** Logistic regression analyses of association between *TCF7L2* rs7903146C>T and rs290481 T>C polymorphisms and risk of T2DM

Genotype	Cases (n=502)		Controls (n=782)		Crude OR (95%CI)	*P*	Adjusted OR^a^ (95%CI)	*P*
	n	%	n	%				
*TCF7L2* rs7903146C>T								
CC	478	96.18	740	94.63	1.00			
CT	19	3.82	41	5.24	0.71(0.41-1.24)	0.227	0.68(0.39-1.18)	0.170
TT	0	0	1	0.13	-	-	-	-
CT+TT	19	3.83	42	5.37	0.70(0.40-1.22)	0.208	0.67(0.38-1.17)	0.160
CC+CT	497	100.00	781	99.87	1.00		1.00	
TT	0	0	1	0.13	-	-	-	-
T allele	19	1.91	43	2.75				
*TCF7L2* rs290481 T>C								
TT	172	34.61	313	40.23	1.00			
TC	246	49.50	341	43.83	**1.29(1.01-1.65)**	**0.042**	**1.32(1.03-1.69)**	**0.030**
CC	79	15.90	124	15.94	1.14(0.82-1.60)	0.443	1.15(0.82-1.62)	0.413
TC+CC	325	65.39	465	59.77	**1.27(1.01-1.61)**	**0.044**	**1.30(1.02-1.64)**	**0.032**
TT+TC	418	84.10	654	84.06	1.00		1.00	
CC	79	15.90	124	15.94	1.00(0.73-1.36)	0.984	1.00(0.73-1.36)	0.979
C allele	404	40.64	589	37.85				

^a^ Adjusted for age, sex, BMI, alcohol use and smoking status.

Bold values are statistically significant (*P* <0.05)

### Association of *TCF7L2* rs290481 T>C polymorphism with T2DM in different stratification groups

Table 4 summarizes the genotype frequencies of *TCF7L2* rs290481 T>C polymorphism in the subgroup analyses. In female subgroup, after adjustment for age, body mass index (BMI), smoking status and alcohol use by logistic regression analysis, the *TCF7L2* rs290481 CC genotype were associated with an increased risk of T2DM compared with the *TCF7L2* rs290481 TT and TC/TT genotypes [CC vs. TT: adjusted OR = 2.05, 95% CI 1.12–3.76, *P* = 0.020 and CC vs. TC/TT: adjusted OR = 1.83, 95% CI = 1.05–3.21, *P* = 0.034 (Table 4)]. In ≥65 years subgroup, after adjustment for gender, BMI, smoking status and alcohol use by logistic regression analysis, the *TCF7L2* rs290481 TC/CC and TC genotypes increased the risk of T2DM compared with the *TCF7L2* rs290481 TT genotype [TC vs. TT: adjusted OR = 1.42, 95% CI 1.01–2.01, *P* = 0.047 and TC/CC vs. TT: adjusted OR = 1.41, 95% CI = 1.01–1.95, *P* = 0.042 (Table 4)]. In never drinking subgroup, after adjustment for gender, age, BMI and smoking status by logistic regression analysis, the *TCF7L2* rs290481 TC/CC and TC genotypes increased T2DM risk compared with the *TCF7L2* rs290481 TT genotype [TC vs. TT: adjusted OR = 1.33, 95% CI 1.02–1.74, *P* = 0.034 and TC/CC vs. TT: adjusted OR = 1.32, 95% CI = 1.03–1.70, *P* = 0.030 (Table 4)]. In BMI < 24 kg/m^2^ subgroup, after adjustment for gender, age, smoking status and alcohol use by logistic regression analysis, the *TCF7L2* rs290481 TC/CC and TC genotypes increased the risk of T2DM compared with the *TCF7L2* rs290481 TT genotype [TC vs. TT: adjusted OR = 1.50, 95% CI 1.04–2.17, *P* = 0.031 and TC/CC vs. TT: adjusted OR = 1.49, 95% CI = 1.05–2.12, *P* = 0.026 (Table 4)].

**Table 4:** Stratified analyses between *TCF7L2* rs290481 T>C polymorphism and T2DM risk by sex, age, smoking status, alcohol consumption and BMI

Variable	(case/control)^a^				Adjusted OR^b^ (95% CI); *P*				
	TT	TC	CC	TC /CC	TT	TC	CC	TC /CC	CC vs. (TC/TT)
Sex									
Male	114/201	167/223	48/95	215/318	1.00	1.33 (0.98-1.81); *P*: 0.071	0.90 (0.59-1.37); *P*: 0.621	1.21 (0.91-1.62); *P*: 0.198	0.77 (0.53-1.13); *P*: 0.185
Female	58/112	79/118	31/29	110/147	1.00	1.27 (0.83-1.96); *P*: 0.277	**2.05 (1.12-3.76);** ***P*: 0.020**	1.47(0.98-2.22); *P*: 0.064	**1.83 (1.05-3.21);** ***P*: 0.034**
Age (years)									
<65	80/153	112/175	32/58	144/233	1.00	1.21 (0.84-1.74); *P*: 0.306	1.05 (0.63-1.76); *P*: 0.857	1.19 (0.84-1.68); *P*: 0.331	0.95 (0.59-1.53); *P*: 0.833
≥65	92/160	134/166	47/66	181/232	1.00	**1.42 (1.01-2.01);** ***P*: 0.047**	1.27 (0.80-2.01); *P*: 0.313	**1.41 (1.01-1.95);** ***P*: 0.042**	1.05 (0.69-1.60); *P*: 0.810
Smoking status									
Never	114/217	160/239	57/82	217/321	1.00	1.29 (0.96-1.75); *P*: 0.097	1.36 (0.90-2.05); *P*: 0.143	1.31 (0.98-1.75); *P*: 0.064	1.18 (0.81-1.71); *P*: 0.391
Ever	58/96	86/102	22/42	108/144	1.00	1.35 (0.87-2.10); *P*: 0.188	0.79 (0.42-1.47); *P*: 0.456	1.24 (0.81-1.89); *P*: 0.315	0.69 (0.39-1.22); *P*: 0.198
Alcohol use									
Never	156/280	219/297	74/110	293/407	1.00	**1.33 (1.02-1.74);** ***P*: 0.034**	1.22 (0.85-1.74); *P*: 0.282	**1.32 (1.03-1.70);** ***P*: 0.030**	1.05 (0.75-1.45); *P*: 0.791
Ever	16/33	27/44	5/14	32/58	1.00	1.25 (0.58-2.70); *P*: 0.564	0.73 (0.22-2.40); *P*: 0.606	1.18 (0.56-2.51); *P*: 0.662	0.65 (0.22-1.96); *P*: 0.445
BMI (kg/m^2^)									
<24	65/175	108/190	35/69	143/259	1.00	**1.50 (1.04-2.17);** ***P*: 0.031**	1.36 (0.83-2.24); *P*: 0.223	**1.49 (1.05-2.12);** ***P*: 0.026**	1.08 (0.69-1.70); *P*: 0.720
≥24	107/138	138/151	44/55	182/206	1.00	1.17 (0.83-1.64); *P*: 0.381	0.99 (0.62-1.59); *P*: 0.957	1.13 (0.82-1.57); *P*: 0.447	0.92 (0.59-1.42); *P*: 0.692

^a^ The genotyping was successful in 502 (99.00%) T2DM cases, and 782 (99.49%) controls for *TCF7L2* rs290481 T>C;

^b^ Adjusted for age, sex, BMI, alcohol use and smoking status (besides stratified factors accordingly) in a logistic regression model;

Bold values are statistically significant (*P* <0.05)

### SNP haplotypes

Using SHESIS program [14], we constructed *TCF7L2* haplotypes. We found there was no significant difference in *TCF7L2* haplotype distribution among T2DM cases and controls (Table 5).

**Table 5:** *TCF7L2* haplotype frequencies (%) in cases and controls and risk of type 2 diabetes

Haplotypes	Cases (n=1004)	Controls (n=1564)	Crude OR (95% CI)	*P*
	n (%)	n (%)		
T_rs290481_C_rs7903146_	575(58.32)	933(59.96)	1.00	
C_rs290481_C_rs7903146_	392(39.76)	580(37.28)	1.10(0.93-1.29)	0.292
T_rs290481_T_rs7903146_	13(1.32)	34(2.19)	0.62(0.32-1.19)	0.170
C_rs290481_T_rs7903146_	6(0.61)	9(0.58)	1.08(0.38-3.06)	1.000

### Relationship of *TCF7L2* rs290481 T>C polymorphism with biochemistry characteristics

Biochemistry characteristics of T2DM cases may be influenced by diet treatment and pharmacotherapy. Thus, in this stage, we assessed the relationship of *TCF7L2* rs290481 T>C polymorphisms with the biochemistry characteristics in controls. The results are shown in Table 6. We found that *TCF7L2* rs290481 T>C polymorphism significantly increased the level of fasting plasma glucose (FPG) and high-density lipoprotein cholesterol (HDL-C) in controls. When the FPG level of *TCF7L2* rs290481 TT genotype was used as the reference, FPG level of the rs290481 CC genotypes significantly increased [CC vs. TT: *P* = 0.029(Table 6)]. When the HDL-C level of *TCF7L2* rs290481 TT genotype was used as the reference, HDL-C level of the rs290481 TC/CC genotypes significantly increased [TC/CC vs. TT: *P* = 0.030(Table 6)].

**Table 6:** Associations of the *TCF7L2* rs290481 T>C genetic variants with biochemistry characteristics in healthy controls

Genotype	Controls (n=782)		FPG (mmol/L)	*P*	Total cholesterol (mmol/L)	*P*	Triglyceride (mmol/L)	*P*	HDL-C (mmol/L)	*P*	LDL-C (mmol/L)	*P*
	n	%										
TT	313	40.23	5.11±0.49	1.0	4.86±1.13	1.0	1.60±1.06	1.0	1.27±0.36	1.0	3.13±0.91	1.0
TC	341	43.83	5.13±0.50	0.627	4.88±0.95	0.794	1.53±0.85	0.349	1.32±0.38	0.064	3.13±0.74	0.966
CC	124	15.94	**5.22±0.43**	**0.029**	4.96±0.93	0.359	1.50±0.98	0.341	1.34±0.36	0.062	3.19±0.80	0.477
TC+CC	465	59.77	5.15±0.48	0.224	4.90±0.95	0.560	1.52±0.89	0.258	**1.33±0.37**	**0.030**	3.15±0.76	0.743

FPG: fasting plasma glucose;

HDL-C, high-density lipoprotein cholesterol;

LDL-C, low-density lipoprotein cholesterol;

Bold values are statistically significant (*P* <0.05)

### The power of the present study (α= 0.05)

For *TCF7L2* rs290481 T>C polymorphism, the power value was 0.522 in TC *vs.* TT and 0.518 in TC/CC *vs.* TT. In the subgroup analyses, the results of power value were: 0.657 in CC *vs.* TT and 0.586 in CC *vs.* TC/TT among female subgroup; 0.518 in TC *vs.* TT and 0.551 in TC/CC *vs.* TT among ≥65 years subgroup; 0.569 in TC *vs.* TT and 0.597 in TC/CC *vs.* TT among never smoking subgroup, and 0.579 in TC *vs.* TT and 0.611 in TC/CC *vs.* TT among never drinking subgroup.

## DISCUSSION

In the past decades, diabetes mellitus has become a public health concern worldwide. The characteristics of diabetes mellitus are the increased level of glucose, metabolism disturbance, and absolute and/or relative insulin resistance (IR). T2DM is one of the most common metabolic disorders in which a number of hereditary and environmental factors may contribute to its etiology. In this study, we focused on the association of energy metabolism and insulin-sensibility relative gene (*TCF7L2*) polymorphisms with T2DM susceptibility. We found *TCF7L2* rs290481 T>C was associated with the development of T2DM and the increased level of FPG.

Transcription factor 7-like 2 (*TCF7L2*), a transcription factor, is expressed in pancreatic β-cells [3] and plays an important role in maintaining glucose homeostasis. It is well known that *TCF7L2* gene is a candidate susceptibility gene of T2DM [3, 15, 16]. In this study, we selected two functional SNPs in *TCF7L2* gene to explore the potential relationship of susceptibility to T2DM. *TCF7L2* rs290481 T>C polymorphism is a locus in intron. Several recent reports suggested that *TCF7L2* rs290481 T>C might play an important role in diabetes mellitus. Delgado-Lista *et al.* reported that this SNP was correlated with differences in insulin secretion, blood lipids, coagulation and blood pressure in metabolic syndrome (an entity often preceding T2DM) cases [17]. Another study indicated the rs290481 C allele near the 3’ end of *TCF7L2* gene was associated with an increased steady-state plasma glucose concentration, a higher 2-h post-challenge glucose and lower waist circumference in Chinese population [18]. However, a previous case-control study focused on the relationship of *TCF7L2* rs290481 T>C polymorphism with gestational diabetes mellitus (GDM) in a Chinese Han population and suggested that this polymorphism was not a potential clinical value for the prediction of GDM [19]. In the present study, we identified a significant association between *TCF7L2* rs290481 T>C polymorphism and the development of T2DM. In addition, the rs290481T→C variant in *TCF7L2* gene promoted the level of FPG. In the future, function of *TCF7L2* rs290481 T>C polymorphism should be further studied to confirm or refute our primary findings.

Some studies reported that carriers of *TCF7L2* rs7903146 T allele have higher susceptibility of T2DM [20]. This phenomenon might be due to impaired insulin secretor and/or insulinotropic effects of incretins rather than an insulin resistance [21, 22]. Recently, several case-control studies focused on the relationship of *TCF7L2* rs7903146 C>T with the development of T2DM in China [7, 10, 11]. The results of a meta-analysis also indicated that *TCF7L2* rs7903146 C>T variants might confer an increased risk to T2DM in Chinese population [13]. As shown in Table 4, we failed to establish a significant association between the *TCF7L2* rs7903146 C>T variants and T2DM in Chinese Han individuals, which was not similar to results of the previous meta-analysis. The low penetrance of T2DM susceptibility from *TCF7L2* rs7903146 C>T variants might be diluted by the interaction of gene-gene and gene-environment. Considering the complexity of T2DM etiology, other important genetic and environmental factors should not be ignored.

Like all epidemiological case–control studies, our investigation had several limitations. Firstly, T2DM cases and healthy controls were enrolled from two local hospitals in Eastern China and might therefore not be well-representative of the general Chinese population; the familial T2DM history of the participants was not considered, this bias might lead to spurious findings. Secondly, in this study, we only investigated two functional SNPs in *TCF7L2* gene, which might not present an extensive view of the genetic variability of *TCF7L2*. In the future, further studies with larger sample size are needed to identify the potential genetic mechanism of T2DM by a comprehensive fine-mapping study. Thirdly, the number of participants included was moderate, the statistical power of the present study might be limited. Finally, environmental risk factors may be different between Eastern Chinese Han population and others. Thus, the risk of T2DM is likely to be affected by gene–gene and gene–environment interactions with different degrees. The association of *TCF7L2* polymorphism with T2DM risk may differ in different ethnic subgroups.

Our findings suggest that *TCF7L2* rs290481 T>C polymorphism is associated with T2DM susceptibility in Eastern Chinese Han population and links to variations in FPG level. In addition, these relationships are more pronounced in female, older subjects and never drinking and BMI < 24 kg/m^2^ subgroups. A comprehensive fine-mapping study with functional investigation is needed to confirm the potential correlation between *TCF7L2* polymorphisms and the development of T2DM.

## MATERIALS AND METHODS

### Subjects

The patient group consisted of 502 subjects with T2DM was diagnosed at the Department of Endocrinology in the Affiliated People's Hospital of Jiangsu University and Fujian Medical University Union Hospital. Patients with a confirmed diagnosis following the World Health Organization 1999 guidelines of T2DM were enrolled to participate in this case-control study: a FPG level of ≥ 7.0 mmol/L and/or a 2h postprandial plasma glucose (PPG) ≥ 11.1 mmol/L after an oral glucose tolerance test [23]. The healthy control group consisted of 782 subjects who attended health check in these hospitals. The major including criteria for controls were: (a) no history of T2DM, (b) PPG < 7.8 mmol/L and (c) fasting plasma glucose (FPG) < 6.1 mmol/L [24]. T2DM patients and healthy controls were recruited between October 2014 and May 2016 consecutively. There was no significant difference in the distribution of sex or age between these two groups. Our study was approved by the Ethics Committee of Jiangsu University and Fujian Medical University, and all participants signed a written informed consent. All subjects were interviewed by two experienced doctors and answered a questionnaire regarding demographic variables and risk factors. Systolic blood pressure, diastolic blood pressure, weight and height were measured. Biochemistry characteristics [e.g. total cholesterol, HDL-C, low-density lipoprotein cholesterol (LDL-C), serum triglycerides and FPG) were also tested. BMI was assessed as weight (kg) divided by height (meter) squared. BMI ≥ 24 was used as the criteria for overweight and obesity in Chinese adults [25, 26].

### DNA extraction and genotyping

Peripheral blood sample was collected in EDTA vacutainer tubes for the extraction of genomic DNA using the Promega Genomic DNA Purification Kit (Promega, Madison, USA). *TCF7L2* rs7903146C>T and rs290481 T>C polymorphisms were determined by the SNPscan^TM^ genotyping assay (Genesky Biotechologies Inc., Shanghai, China), which is a double ligation and multiplex fluorescence PCR [27]. Fifty-two DNA samples were randomly selected and tested again for quality control. The genotypes of *TCF7L2* rs7903146C>T and rs290481 T>C polymorphisms were confirmed.

### Statistical analysis

Continuous variables [e.g. age, BMI, FPG, total cholesterol, triglyceride, HDL-C and LDL-C levels] are expressed as means ±standard deviation (SD). Comparisons between T2DM patients and healthy controls were performed with Student's t-test. The categorical variables (e.g. *TCF7L2* genotypes, sex, age, smoking status, drinking and BMI) were compared with Chi-square test (*χ*^2^). Deviations from the HWE in controls were determined by an internet-based calculator (http://ihg.gsf.de/cgi-bin/hw/hwa1.pl). The relationship of *TCF7L2* rs7903146C>T and rs290481 T>C polymorphisms with T2DM risk was evaluated by odds ratios (ORs) and the corresponding 95% confidence intervals (CIs). Multivariate linear regression was harnessed to determine the association of *TCF7L2* rs7903146C>T and rs290481 T>C polymorphisms with quantitative traits. All multivariate linear regression analyses were adjusted for age, sex, alcohol use, smoking status and BMI. Analysis was performed with SAS software Version 9.4 (SAS Institute, Cary, NC). A *P* < 0.05 with two-tailed test was defined as the criterion of statistical significance. In this study, the Power and Sample Size Calculator software (http://biostat.mc.vanderbilt.edu/twiki/bin/view/Main/PowerSampleSize) was used to calculate the power value (α = 0.05). SHESIS software (Bio-X Inc., Shanghai, China,http://analysis.bio-x.cn/myAnalysis.php), an online haplotype calculator, was used to construct *TCF7L2* haplotypes [14].
